# Rodent Models of Optic Neuritis

**DOI:** 10.3389/fneur.2020.580951

**Published:** 2020-11-03

**Authors:** Yael Redler, Michael Levy

**Affiliations:** ^1^Department of Neuro-Ophthalmology, Massachusetts Eye & Ear Infirmary/Harvard Medical School, Boston, MA, United States; ^2^Department of Neurology, Massachusetts General Hospital/Harvard Medical School, Boston, MA, United States

**Keywords:** optic neuritis, rodent models, experimental autoimmune encephalomyelitis, multiple sclerosis, demyelination

## Abstract

Optic neuritis (ON) is an inflammatory attack of the optic nerve that leads to visual disability. It is the most common optic neuropathy affecting healthy young adults, most commonly women aged 20–45 years. It can be idiopathic and monophasic or as part of a neurologic disease such as multiple sclerosis with recurrence and cumulative damage. Currently, there is no therapy to repair the damage from optic neuritis. Animal models are an essential tool for the understanding of the pathogenesis of optic neuritis and for the development of potential treatment strategies. Experimental autoimmune encephalomyelitis (EAE) is the most commonly used experimental rodent model for human autoimmune inflammatory demyelinating diseases of the central nervous system (CNS). In this review, we discuss the latest rodent models regarding optic neuritis, focusing on EAE model, and on its recent achievements and developments.

## Introduction

### Optic Neuritis

Optic neuritis is an inflammatory demyelinating disorder of the optic nerve. The typical form of optic neuritis is idiopathic or associated with multiple sclerosis (MS). Atypical forms of optic neuritis can occur in association with other inflammatory disorders or due to infections, immune-stimulating medications, and paraneoplastic disorders.

The prevalence of optic neuritis is estimated to be as high as 115/100,000 depending upon geography and ethnicity (1). The incidence is highest in populations located at higher latitudes such as northern USA, Europe, and Australia compared with geographic locations closer to the equator (2). There is a female preponderance with 3:1 ratio, with most patients 20–45 years old (3).

According to the involved site, optic neuritis can be classified as retrobulbar optic neuritis, affecting any part of the optic nerve behind its entry to the eyeball. This is the most common form of optic neuritis, and on examination, the disk often appears normal. Optic neuritis that includes inflammation of the optic disk is known as papillitis, which is visible on examination as hyperemia, swelling of the disk, blurring of disk margins, and distended veins (4). An afferent pupillary defect in the affected eye is usually detectable.

The common clinical presentation of a patient with optic neuritis is unilateral visual acuity loss, visual field loss, color vision deficits, decreased contrast, and brightness sense. There is also periocular pain precipitated by eye movements that may precede the visual loss by a few days. The extent of visual function damage may vary significantly according to the etiology of the optic neuritis. In typical optic neuritis and optic neuritis associated with MS, visual acuity loss is moderate, conversely, optic neuritis associated with neuromyelitis optica spectrum disorder (NMOSD) or myelin oligodendrocyte glycoprotein (MOG) often presents with severe vision loss (5, 6).

Diagnostic investigations include MRI, visual evoked potentials (VEP), and cerebrospinal fluid (CSF) examination. MRI is performed to characterize the location and extent of inflammation and to rule out other etiologies such as multiple sclerosis. VEPs measure optic nerve function and may be useful when anatomic studies by MRI are equivocal. Degeneration of retinal ganglion cells (RGC) and retinal thinning which correlate with measures of persistent visual dysfunction after optic neuritis is demonstrated by optical coherence tomography (OCT) (7, 8).

Treatment with high-dose corticosteroids shortens the period of acute visual dysfunction but does not affect the final visual outcome in typical optic neuritis (9). Atypical forms can necessitate prolonged immunosuppressive regimens. Recently, therapies to promote neuroprotection and remyelination have been investigated (10, 11) along with immunosuppressive therapies for autoimmune processes to prevent recurrence of immune-mediated damage (12).

## Rodent Models of Optic Neuritis

Animal models are essential for the understanding of etiology and pathogenesis of immune-mediated processes and to develop therapeutic strategies which eventually will lead to effective treatments for human diseases. Many studies have been conducted in order to understand the pathophysiological mechanisms of optic neuritis using different models.

### Experimental Autoimmune Encephalomyelitis Models

The EAE model is the most commonly used experimental rodent model for human autoimmune inflammatory demyelinating diseases of the CNS. EAE is a complex system of interaction between multiple immunological and neuropathological mechanisms leading to inflammation, demyelination, axonal loss, and gliosis. The regulatory mechanisms of resolution of inflammation and remyelination also occur in EAE.

In EAE, the optic nerve lesions are, in most cases, a part of the whole CNS disease process which includes the brain and the spinal cord. There are subtypes that are found to be more associated with optic nerve involvement. For example, in the dark agouti rat model, acute optic nerve inflammation manifests earlier than spinal cord inflammation (PMID: 31267597) whereas in C57BL6 mice, optic nerve inflammation occurs chronically along with inflammation in the rest of the CNS (PMID: 29903027).

### Induction Phase

EAE is initiated by introducing a specific CNS antigen, such as MOG, myelin basic protein (MBP), or proteolipid protein (PLP), in the context of an inflammatory stimulus to induce encephalomyelitis. It is typically induced by either active immunization with myelin-derived proteins introduced into the CNS, emulsified in complete Freund's adjuvant (CFA), which contains heat-inactivated *Mycobacterium tuberculosis*. CFA enhances peripheral immune response by promoting Th1 response and increases blood-brain barrier (BBB) permeability (13). Pertussis toxin (PTX) which is administered in the process as well has been suggested to also modulate the blood-brain barrier and the immunological responsiveness.

Passive immunization, by adoptive transfer of activated myelin-specific CD4+ T lymphocytes (14–16), allows EAE to develop faster, without an adjuvant (17). Typically 10–14 days after initiation, the disease is manifested by a variety of clinical and pathological reactions. The extent and location of inflammation and demyelination is variable according to specific antigen introduced, rodent species, strain, age, and gender. As a consequence, an acute or chronic-relapsing inflammatory demyelinating autoimmune disease is acquired (18, 19).

### Inflammatory Phase

The inflammatory process in the CNS is first indicated by activated microglia, local macrophages, and peripheral T lymphocytes. Activated T cells undergo maturation and clonal expansion; later, they differentiate into effector cells and migrate through the blood circulation to breach the BBB. Adhesion molecules are expressed on endothelial cells in CNS microvasculature, allowing activated T cells to bind to these molecules and penetrate the endothelium. To further penetrate the subendothelial basement membrane which is mainly composed of type IV collagen, T cells utilize matrix-degrading enzymes (20). Once they enter the CNS, T cells recognize antigen presented locally and become reactivated, enhance inflammation, and continuously recruit other cells (21). Secretion of cytokines and other proinflammatory mediators further exacerbate the inflammatory milieu which recruits additional immune cells into the CNS and culminates in demyelination (22, 23).

### Demyelination and Axonal Loss

Myelin loss leads to a disruption of axonal function, and axons may eventually die back depending on the severity and persistence of the inflammatory response. Axons are vulnerable to damage by inflammatory cytokines, enzymes, and nitric oxide which are expressed by activated immune cells during the inflammatory process and cause direct cytotoxic effects. Axons that survive demyelination when the inflammation resolves may become remyelinated; however, in most EAE phenotypes, inflammatory damage leads to neuronal cell death, axonal loss, and gliosis (24–26). Irreversible axonal damage is seen by retinal nerve fiber layer (RNFL) thinning (27–29).

The clinical evaluation of the disease progression can be done in several ways. Due to the fact that active EAE is an ascending progressive disease that progresses into paresis, it initially affects rodent tail tone, followed by limb motor deficits. Daily scoring of these parameters provides information regarding spinal cord inflammation and demyelination (30). The optic neuritis progression can be assessed by examining the visual response to specific stimuli such as OKN response or by assessing the response to hand movements in front of the rodent, and in some studies, rodents who did not escape when a sharp-pointed object held in front of their eyes were thought to be blind (31). Optic neuritis was also assessed by RNFL thickness using an OCT and retinal ganglion cell count (32). Other ways to assess the optic nerve inflammation and demyelination are through histology of the optic nerve (33).

### Other Rodent Models of Optic Neuritis

Engineering a new MOG-specific TCR transgenic mouse, the 2D2 mouse made by Bettelli et al. in 2003 produces transgenic T cells with receptors capable of recognizing MOG presented by MHCII (34). Approximately 30% of these mice will develop spontaneous optic neuritis; when immunized with subclinical levels of MOG peptide, up to 56% developed histological evidence of EAE. When they were fully immunized, 80% developed optic neuritis. It was assumed that the specificity of inflammatory demyelination is related to the significantly higher levels of MOG in the optic nerve than the spinal cord.

Neuromyelitis optica (NMO) is an inflammatory demyelinating disease involving the optic nerves and spinal cord. The damage is caused due, in part, to autoantibodies against aquaporin-4, a water channel on astrocytic foot processes at the blood-brain barrier. There are several animal models of NMO that have been developed (35). By crossing 2D2 mice with MOG-specific Ig heavy-chain knock-in mice (IgHMOG mice), severe inflammatory demyelination involving the optic nerves and the spinal cord developed in about 60% of these mice. Immunoglobulin class switching was observed, indicating that B and T lymphocyte cooperation play a role in induction of autoimmune processes.

Several studies showed that passive transfer alone of the aquaporin-4 antibody from patients with neuromyelitis optica is insufficient to reproduce the disease in rodents unless extremely high levels are infused. In the context of EAE with myelin-targeted T cells or even after an injection of Freund's adjuvant, the antibody can reach its target in the CNS and contribute to inflammatory demyelination (36–39). Direct injection of the antibody into the brain along with human complement can also induce inflammatory demyelination (40). More recently, a mouse model of NMO could be recapitulated by adoptive transfer of T cells reactive to aquaporin-4 (41, 42).

## Optic Neuritis Pathomechanism

In 1977, an experimental model for acute allergic optic neuritis was induced in guinea pigs. They exhibited two distinct clinical patterns: “retrobulbar optic neuritis” with normal fundus and “neuroretinitis” with hyperemia and swelling of the disk and retinal edema. Histopathology of the “retrobulbar neuritis” revealed that some of them had brain involvement without any involvement of the optic nerve, while others involved the retrobulbar portion of the optic nerve and chiasm with multiple foci of demyelination. In the neuroretinitic animals, the lesions were localized behind the lamina cribrosa and had an appearance characteristic of papilledema (43).

Due to a unique anatomic structure, where the anterior part of the optic nerve within the lamina cribrosa is unmyelinated and supported by modified astrocytes, a barrier between the optic nerve and the retina restricts the inflammation to the optic nerve during ON, and prevents retinal inflammation as well (44, 45). In New Zealand albino rabbits, the axons of the nerve fiber layer are myelinated over a long portion within the retina. Extensive inflammatory lesions were observed in the myelinated fibers within the retina following sensitization with bovine myelin and adjuvant (46).

Theories regarding specific proteins playing a critical role in the mechanism of EAE optic neuritis have been proposed. Lipocalin-2, a protein that regulates diverse cellular processes, is expressed and secreted by microglia and astrocytes due to inflammatory stimuli in the central nervous system (47). Lipocalin-2 is a pro-inflammatory activator of T cells during EAE development and progression (48). Mice deficient in lipocalin-2 showed a significant reduction of demyelination, inflammatory infiltration, and gliosis in the optic nerve in EAE (49). Another area of specific investigation was the site of vulnerability in the optic nerve head where an incomplete blood-brain barrier allows partial access to the immune-privileged CNS. Using the expression of αB-crystallin, which is a heat-shock protein expressed as an early stress response in oligodendrocytes, one of the earliest targets of EAE is the optic nerve head associated with IgG deposition suggesting that partial immune access at this area of the CNS may explain its specific vulnerability in EAE (50).

Astrocytes in the optic nerve are known to play a pivotal role in neuroinflammatory processes in the CNS. In the human disease NMOSD, the astrocytic water channel aquaporin-4 is the naïve target of an aberrant immune response, especially in the optic nerve (51). The role of astrocytes in typical optic neuritis was studied by characterizing the astrocyte-specific transcriptome in EAE optic neuritis. Their results showed a significant increase in the proinflammatory-complement cascade, especially complement component 3 (C3) and a decrease in factors of the cholesterol biosynthesis pathways involved in remyelination. Interestingly, these changes in astrocytes as well as increased axonal loss were greater in EAE females vs. males (32).

A study suggested that dietary supplementation with a balanced mixture of fatty acids (FAs) including omega 3 and omega 6, efficiently limit inflammation and prevent RGC degeneration, demonstrating a neuroprotective effect in EAE models (52). Further study investigating the mechanisms underlying the anti-inflammatory effects of fatty acids found that they shift macrophage polarization from the M1 inflammatory phenotype, which releases proinflammatory cytokines, leading to tissue damage in the CNS, toward the anti-inflammatory M2 phenotype associated with resolving inflammation and tissue repair (53).

## Medications

### Antioxidants Therapies

Reactive oxygen species (ROS) are formed as byproducts in a variety of biochemical reactions. When generated in excess or not appropriately regulated, ROS may cause cellular damage and tissue injury. During an inflammatory process, activated immune cells release ROS, leading to oxidative stress and tissue damage and causing demyelination and axonal destruction (54). Reduction in oxidative damage is an important therapeutic strategy. In recent years, many studies are focused on antioxidant-based treatment for neuro-inflammatory diseases (55).

Alpha lipoic acid (ALA) is a naturally occurring antioxidant which has recently been investigated for its neuroprotective capacities during inflammation (56). It has been demonstrated to reduce the rate of brain atrophy in progressive MS (57), and it is highly effective at suppressing and treating EAE. Mice that received ALA experienced a dose-dependent reduction in cumulative disease scores (58). In another study, it was demonstrated that mice with EAE that received ALA had a dramatic reduction in axonal injury compared with saline-treated mice (59). However, another study demonstrated that therapeutic treatment with ALA attenuates the clinical disability and improves the survival of RGCs in the EAE model while prophylactic ALA therapy is capable of preserving visual function and prevention of thinning of the inner retinal layer (60). A clinical trial that tried to determine whether lipoic acid is neuroprotective in acute optic neuritis in humans did not conclude that 6 weeks of oral LA supplementation treatment after acute optic neuritis was neuroprotective. It is safe though and well tolerated (61). The antioxidant idebenone is beneficial in two neurological disorders caused by mitochondrial alterations: Friedreich's ataxia and Leber's hereditary optic neuropathy. In a study using an EAE model, idebenone-treated mice showed no improvement in inflammation, demyelination, or axonal damage. It failed to affect disease when applied preventively or therapeutically (62). Bilirubin has previously been demonstrated to be a potent antioxidant *in vitro* (63). When administered to rats before the onset of EAE, it was shown to have a protective effect on BBB from increasing permeability due to ROS damage and by that, preventing invasion of inflammatory cells into CNS during inflammation. In rats that were treated after induction of EAE, bilirubin did not reduce the degree of inflammation or cytokine expression but did demonstrate clinical improvement (64). A study aimed to investigate the effect of melatonin demonstrated a neuroprotective effect against EAE, by suppressing the progression and lymphocytic infiltration. This effect was probably related to the decrease of the levels of oxidative stress (65). The amino acid acetyl-l-carnitine (ALCAR) was evaluated for its effects when used alone or together with corticosteroids for treatment of EAE. A combination of the two demonstrated an antioxidant, antiapoptotic, and immunosuppressive effect. It also improved the clinical outcome when compared with the untreated group or corticosteroid treatment alone (66).

Unfortunately, studies suggesting antioxidative-based treatment for optic neuritis that showed benefit in the EAE model, did not progress to clinical trial in humans, and if so, did not show any benefit for the treatment in these cases.

### Neuroprotective Therapies

Optic neuritis may lead to permanent visual loss mediated by RGC damage. The goal of neuroprotection is to preserve axon structures and function; it is highly important given the poor regenerative capacity of neurons. The specific mechanism and timing of the RGC damage during the disease process are crucial for understanding the damage and how to prevent it. In a study aimed to determine whether axonal injury due to inflammation mediates apoptotic death of RGCs, EAE was induced followed by demyelination with significant axonal loss which was followed by loss of RGC. It was suggested that inflammatory cell infiltration mediates demyelination and leads to direct axonal injury. RGCs die by an apoptotic mechanism triggered by axonal injury (67). Another study demonstrated DNA degradation and activation of caspase-3 in RGCs of EAE-induced rats. This indicates that cell death of RGCs is apoptotic (68). Potential neuroprotective therapies to prevent permanent RGC loss from optic neuritis need to be initiated prior to axonal injury to preserve neuronal function. A study that examined potential neuroprotective effects in optic neuritis by SRT647 and SRT501, activators of SIRT1, an enzyme involved in cellular stress resistance and survival, demonstrated that SIRT1 activation prevents RGC loss in optic neuritis even in the presence of active inflammation, suggesting that their neuroprotective effects will be additive to other immunomodulatory treatments (69).

High-dose intravenous corticosteroids are the standard treatment for acute optic neuritis. The optic neuritis treatment trial (ONTT) demonstrated that intravenous methylprednisolone followed by oral prednisone helps recovery of visual acuity and results in slightly better vision at 6 months. Oral prednisone alone is not an effective treatment. It increases the risk of recurrent episodes of optic neuritis (9). However, a study demonstrated a proapoptotic effect of the RGCs induced by methylprednisolone treatment for acute optic neuritis, although inflammatory infiltration of the optic nerve was reduced (70).

Brimonidine (BMD) is a selective α2-adrenergic receptor agonist that is used clinically for the treatment of glaucoma. Topical administration of BMD at 0.2% was shown to result in vitreous concentration of 2 nM and above, which is known to activate alpha(2)-receptors (71). There have been several studies demonstrating a neuroprotective effect of BMD. One of them evaluated BMD effect on retinal degeneration during optic neuritis in EAE, showing a suppression of the significant reduction in the number of RGCs, indicating the functional significance of the neuroprotective effect of BMD (72).

Various mechanisms for neuroprotection by brimonidine have been suggested, including brain-derived neurotrophic factor (BDNF) activation in the retinal ganglion cells, glutamate inhibition, suppression of excitotoxicity in retinal ganglion cells, stimulating trophic factor release from Müller cells, and cell-survival signal upregulation, as well as apoptosis downregulation (73, 74).

Voltage-gated sodium channels (Nav) are suggested to have a key role in the etiology of EAE by causing axonal degeneration (75). Nav1.6 isoform is a promoter of neuronal degeneration and inflammation in EAE (76), suggesting that it plays a corresponding role in MS and possibly in other degenerative neurological diseases. It was shown that downregulating or blocking Nav1.6 on neuronal cells would be neuroprotective (77).

Phenytoin is a voltage-gated sodium channel blocker used as an antiseizure medication. A study evaluating the effect of phenytoin on axonal degeneration in the optic nerve in EAE, reported that whereas ~50% of optic nerve axons are lost at 27–28 days in untreated EAE, only ~12% of the axons are lost if mice with EAE are treated with phenytoin (78).

### Remyelination-Based Therapies

Myelin is a lipid-rich protective covering that surrounds axons. It provides an electrical isolation that allows fast nerve transmission and metabolic support to neurons. In demyelinating diseases, myelin loss leads to disruption in electrical conduction (79). Following pathological loss of myelin, remyelination, the formation of new myelin sheaths around axons, occurs (80). Myelin is formed by newly differentiated oligodendrocytes (OLs) derived from oligodendrocyte progenitors (OPCs) at the lesion sites. Microglia activation plays a crucial role in promoting oligodendrocyte maturation and effective remyelination (81). Unfortunately, the remyelination process is often inefficient, leading to permanent deficits and dysfunction. Its failure results from the inability of OPCs to successfully generate new mature myelinating oligodendrocytes (82). Many processes have been identified as targets for therapies that enhance the remyelination process in neuro-inflammatory demyelinating diseases.

A major target is lipid metabolism in oligodendrocytes. Lipids account for about 70% of the myelin membrane. A decrease in cholesterol synthesis in astrocytes might affect demyelination of autoimmune disease (83). Cholesterols in the brain must be synthesized locally because peripheral cholesterol does not cross the BBB. It was shown that dietary cholesterol supplements promote the repair of demyelinated lesions. Concomitant with blood-brain barrier impairment, they directly supports oligodendrocyte precursor proliferation and differentiation (84). In the adult brain, cholesterols are synthesized in astrocytes, transported to neurons to make cell membranes and synapses, and to oligodendrocytes to make myelin (85–88). Thus, reduced cholesterol synthesis in astrocytes during an inflammatory process may lead to reduced cholesterol available for neurons and oligodendrocytes, hence limited reparative synaptic plasticity and remyelination.

Normally, there is a balance between the rate of demyelination and remyelination processes. Myelin debris are phagocytosed by microglia; when this balance is disrupted, such as in inflammatory demyelinating diseases, it causes an accumulation of myelin debris that impairs remyelination (89).

Another study hypothesized that the inability to repair the demyelination damage caused by the inflammation during EAE may result from decreased cholesterol synthesis by astrocytes. Upregulation of cholesterol-synthesis gene expression was observed in oligodendrocytes during remyelination (90).

Another study demonstrated an accelerated remyelination after EAE induction by direct lineage analysis and hypothesized that newly formed myelin remains stable during the inflammation process due to the absence of MOG expression in immature myelin. Furthermore, withholding oligodendrocyte differentiation and myelination results in acceleration of remyelination, thus preventing axonal loss and improving functional recovery (91).

## Conclusions

Optic neuritis is an inflammatory demyelinating disorder of the optic nerve with up to 0.1% prevalence at certain regions and potentially devastating visual outcomes. Animal models using the EAE experimental rodent model are essential for the understanding of etiology, pathogenesis of immune-mediated processes and for the development of therapeutic strategies for optic neuritis.

In this review, we discussed the latest rodent models regarding optic neuritis, focusing on the widely used EAE model, on its recent achievements and developments. So far, none of the therapeutic strategies have shown a substantial achievement regarding optic neuritis in the field of neuroprotection and remyelination. Further investigation is required.

## Author Contributions

YR and ML contributed to the writing of the manuscript. Both authors contributed to the article and approved the submitted version.

## Conflict of Interest

ML currently receives research support from the National Institutes of Health. He also received personal compensation for consultation with Alexion, Viela Bio, and Genentech and has served on the scientific advisory boards for Alexion, Genentech, and Viela Bio. The remaining author declare that the research was conducted in the absence of any commercial or financial relationships that could be construed as a potential conflict of interest.
